# Investigation of high-resolution computed tomographic (HRCT) outcomes associated with chronic pulmonary microaspiration (CPM) in Tehran and Zahedan, Iran

**DOI:** 10.4314/ahs.v20i4.22

**Published:** 2020-12

**Authors:** Bahareh Heshmat Ghahderijani, Fatemeh Hosseinabadi, Shahram Kahkouee, Mohamad Kazem Momeni, Samira Salajeghe, Hussein Soleimantabar

**Affiliations:** 1 Department of Radiology, Imam Ali Hospital, Zahedan University of Medical Sciences, Zahedan, Iran; 2 Department of Radiology, Chronic Respiratory Diseases Research Center, National Research Institute of Tuberculosis and Lung Diseases (NRITLD), Shahid Beheshti University of Medical Sciences, Tehran, Iran; 3 Department of Internal Medicine, School of Medicine, Zahedan University of Medical Sciences, Zahedan, Iran; 4 Department of Radiology, Bam University of Medical Sciences, Bam, Iran; 5 Department of Radiology, Emam-Hossein Hospital, Shahid Beheshti University of Medical Sciences, Tehran, Iran

**Keywords:** Imaging, high-resolution computed tomographic, chronic lung microaspiration

## Abstract

**Background:**

In patients with chronic pulmonary microaspiration (CPM) the recognition of high-resolution computed tomographic (HRCT) findings and their pattern is important.

**Objective:**

To investigate the HRCT detections in patients with CPM.

**Materials and Methods:**

This descriptive study enrolled 100 consecutive patients with CPM underwent HRCT of the lungs between 2017 and 2018 in Tehran and Zahedan Hospitals and private centers. The required variables were recorded for each patient with a questionnaire. Subsequently, HRCT was performed and abnormalities were then reported by two radiologists.

**Results:**

Most of patients exhibited bronchial thickening in 33.6% of cases, followed by ground-glass opacity (12.4%), emphysema (11.1%), and bronchiectasis (8.5%). In addition, the most common HRCT findings were found in left lower lobe (LLL) (37.1%), followed by right lower lobe (RLL) (35.9 %), right upper lobe (RUL) (6,2%), and left upper lobe (LUL) (6%).

**Conclusion:**

Our data showed the most common findings in HRCT were bronchial thickening ground-glass opacity, emphysema, and bronchiectasis, where these findings was dominantly found in LLL, RLL, RUL, and LUL, indicating its high tendency to dependent areas.

## Introduction

Pulmonary aspirations (PA) are defined as the inhalation of oropharyngeal secretions or gastric matter toward the larynx and lower respiratory system. Aspiration-induced clinical syndrome, such as aspiration and pneumonitis are defined to be related to the kinds and volume of aspirated material, aspiration frequency, and the host's response to aspirated substance 1. Chronic aspiration pneumonia can lead to changes associated with microaspiration or macroaspiration of orogastric content with time. Nearly half of the adults aspirate a small amount of oropharyngeal secretions during sleep, and an increased risk of microaspiration can be associated with other co-morbidities such as scleroderma, cerebrovascular disease and neurodegenerative diseases, an increased risk of microaspiration 1–4. Chronic pulmonary microaspiration (CPM) is one of the complications which have received much attention despite its low incidence (1.4 to 6 per 10,000). Aspiration pneumonia has been accounted for 5–15% of community-acquired pneumonia cases 1,5–8. Aspiration pneumonia is one of the most leading causes of death in subjects with dysphagia, with an estimated case between 300,000 and 6,000,000 annually in the United States 3

Clinical outcomes of CPM are a range of uncomplicated changes to severe respiratory problems and even death. As a matter of fact, regarding the different types of aspirated materials, the range of the disease is ranged from mild pneumonia to severe respiratory distress syndrome, and lack of cardiovascular responses and renal insufficiency. Furthermore, other associated-risk factors will lead to deaths of up to 76%, as well as monetary burden of the disease should be taken into consideration. As a result, its diagnosis is of great importance and it can significantly reduce its complications and treatment costs 9–11. Bronchoalveolar lavage (BAL) is used to evaluate patients suspected of CPM. The use of HRCT has reduced the clinical use of BAL. HRCT specifies special imaging patterns that are associated with CPM. In the past decade, HRCT has been useful in making a specific diagnosis or limiting the differential diagnosis of pulmonary disease 12. Despite the usefulness of HRCT, BAL is still used in cases, where the clinical diagnosis of microaspiration is suspected, but HRCT is normal. In spite of the radiologic diagnosis of CPM in suspected patients, cellular analysis of the BAL is used to confirm HRCT findings and cases without typical clinical symptoms for correct management of patients.

Of course, BAL is not capable of excluding microscopic abnormalities, and in this case HRCT may be helpful. Therefore, considering the overlaps in the efficacy of these two tests, more studies are needed to clarify the efficiency and indication of HRCT. This study was aimed to examine the findings of a HRCT in patients with CPM.

## Methods

### Ethical Committee

The study protocol was approved by the Ethics Committee of Zahedan University of Medical Sciences. All procedures performed in accordance with the ethical standards of the institution and/or national research committee and with the 1964 Helsinki Declaration for human participants

### Patients

In this descriptive study, 100 patients with CPM were enrolled on the basis of the pathology archives in Tehran and Zahedan hospitals and private centers from 2017 to 2018. The inclusion criteria were: i) patients with pulmonary microaspiration, ii) all eligible patients (aged ≥18 years) with CPM based on the histology. In addition, exclusion criteria included i) pregnant women, ii) children, iii) coagulation disorders, iv) cardiovascular instability.

The required variables were recorded for each patient with a questionnaire. Subsequently, HRCT was performed and abnormalities were then reported by two radiologists, who were blinded to the aspiration (e.g., consolidation, collapse, scar, nodule, etc.). According to Cardasis et al. (2014), the observation of lipoid pneumonia, giant cell, foreign material, and granuloma was considered as CPM 11. In other words, microaspiration was approved if this evidence were present.

## Statistical analysis

Frequencies are indicated as percentages for qualitative variables. Correlative analysis was conducted with SPSS software version 24. Differences between the frequencies of location (tendency to involve the dependent areas) were determined using χ2 tests for qualitative variables. A p-value <0.05 was considered to be statistically significant.

## Results

**Fig. 1:**
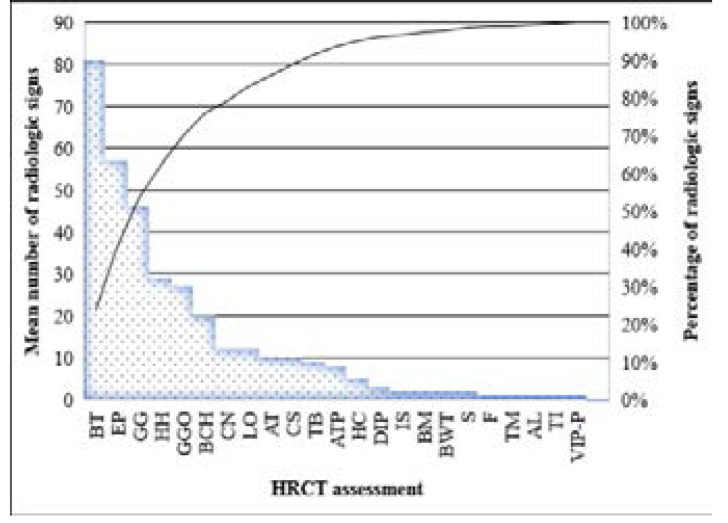
The frequency distribution of HRCT findings in surveyed patients (BT: Bronchial thickening, EP: Emphysema, GG: Ground-glass, HH: Hiatal hernia, GGO: Ground-glass opacities, BCH: Bronchiolectasis, AT: Atelectasis, CN: Centrilobular nodule, LO: Linear opacities, CS: consolidation, TB: Traction bronchiectasis, ATP: Air trapping, HC: Honeycombing, DIP: Desquamative interstitial pneumonia, IS: Interface sign, BM: Bronchomalacia, BWT: Bronchial wall thickening, S: SCAR, F: Fibrosis, TM: Tracheomalacia, AL: Alveolitis, TI: Thickening of interlobular, and VIP-P: VIP pattern)

In this research, a total of 100 patients were contained; the finding presented herein indicated that the most common CT detections were bronchial thickening (33.6%), followed by Ground-Glass Opacity (12.4%), Emphysema (11.1%), and Bronchiectasis (8.5%), (Fig 1).

**Fig. 2:**
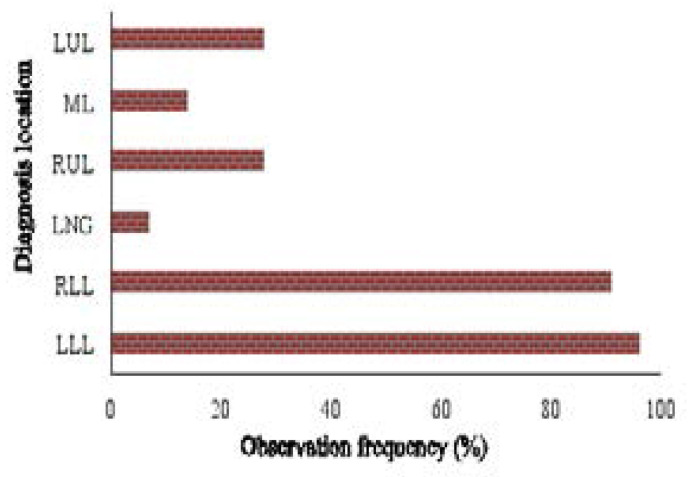
The frequency distribution of findings location in the investigated patients LLL, left lower lobe; LNG, lingular segment; LUL, left upper lobe; ML, middle lobe; RLL, right lower lobe. RUL: right upper lobe

As shown in figure 2, the highest HRCT findings were found to be LLL (37.1%), followed by RLL (35.9%), RUL (6.2%), and LUL (6%). The findings revealed that emphysema was highly more prevalent in RUL, but other findings were more prevalent in LLL and RLL. The analysis of the findings showed that the frequency of location according to the type of HRCT findings was statistically significant (P = 0.0001; Fig 2).

## Discussion

Silent microaspiration has been revealed to be involved in different lung diseases (e.g., chronic bronchiolar and interstitial pulmonary disease, lipoid pneumonia, inflammatory pneumonitis, post-transplantation bronchiolitis, etc.,), 13–17. For instance, silent microaspiration of exogenous lipid can be a causative factor of lipoid pneumonia, leading to a chronic inflammatory pneumonitis and fibrosis 15

HRCT provides is remarkably capable of improving the sensitivity and specificity of clinical and histopathological assessment 18. HRCT can be of great importance in a united method of detection where it can be capable of providing evidence for a various detection and non-invasive definitive detection 19. Despite the radiological diagnosis of CPM in suspected patients, cellular analysis of the BAL is used to confirm the HRCT findings and is also used to manage patients correctly in patients whose clinical symptoms are not typical. Of course, BAL does not rule out the presence of microscopic changes, and in this case HRCT may be capable of showing abnormalities

In the present study, among the patients with aspiration, the most common HRCT patterns include bronchial thickening (33.6%), followed by Ground-Glass Opacity (12.4%), Emphysema (11.1%), and Bronchiectasis (8.5%). As shown in figure 2, the highest HRCT findings were found to be LLL (37.1%), followed by RLL (35.9%), RUL (6.2%), and LUL (6%).

In a study by Scheeren et al., 2016 demonstrated that centrilobular nodules, consolidation, atelectasis, bronchiolectasis, and ground-glass opacities were predominantly found in patients with chronic aspiration as compared to control group, where a remarkable predilection was found for the lower lobes. Aforementioned study indicated that the thickness of the bronchial wall and the air trapping were not significantly different among the groups 20

Another study reported that aspiration pneumonia was mostly observed as a bronchopneumonia pattern and bronchiolitis pattern using CT findings. Moreover, centrilobular nodules, ground-glass attenuation, atelectasis consolidation were among the most common finding of aspiration pneumonia on CT 21. The HRCT findings in our study were more or less consistent with studies in some pattern 7,20,22, although different nonspecific imaging findings have been demonstrated for aspiration^23^. Evidence suggests that that microaspiration cause chronic pneumonitis and long-term pulmonary fibrosis, in addition post-transplantation bronchiolitis, all of which are found in HRCT 1. But in our study, the most common findings of HRCT in patients with CPM included bronchial thickening, ground-glass opacity, emphysema and bronchiectasis. In a study by Elicker et al. (2010), HRCT was the best modalities for microaspiration and ceated a distinct appearance of crazy paving, which is in the form of consolidation with few attenuation and ground-glass opacities 6, which is consistent with our findings. Pereira-Silva and colleagues evaluated^13^ patients and histologic detection of CPM under high-resolution computed tomography (CT) and their main finding was centrilobular nodules and groundglass opacities. Furthermore, branching opacities and small foci of consolidation, septal lines, and bronchiectasis were found to be other common finding in many patients, which is relatively similar to the results of our research. The reason that other findings are not consistent with our research is that HRCT findings by different radiologists can be interpreted differently, and for this reason, we examined the results by two radiologists.

In the study by Oikonomou and Prassopoulos (2013), the following findings were observed in radiological examination of patients with microaspiration: centrilobular nodules, ground-glass opacities, branching opacities, small foci of consolidation, bronchiectasis / bronchioloectasis, reticular interstitial pattern 24, similar findings are also obtained in our study.

The accuracy of HRCT has been previously assessed for the diagnosis of allergic bronchopulmonary aspergillosis in patients with asthma, where Bronchiectasis was found in 95% (42 patients), centrilobular nodules in 93% (41 patients, and mucoid impaction in 29.5 (67%). Diagnosis of microaspiration has been previously evaluated be researchers such as barium swallow, CT scan, and scintigraphy (Gamma scan), 1. Plus, Pereira-Silva et al., 2014 reported that CPM detection has been done by some investigations regarding clinical signs and risk factors^7^, where centrilobular nodules and ground-glass opacities has been demonstrated as CPM outcomes 20 Accumulating evidence indicated Esophagography and CT have been usefully incapable in assessing aspiration disease associated with tracheoesophageal or tracheopulmonary fistula. CT scan is capable of increasing the low accuracy of chest radiography in aspiration diseases along with clinical manifestations and complications^25^. Additionally, CT scan is the modality of choice in detecting asymptomatic aspiration for other indications. Chest CT scan can be effectively useful for detecting early radiographic changes of aspiration, e.g., mild bronchiectasis, pleural thickening and air-trapping, as well as consolidation, and atelectasis 26–29

## Conclusion

In summary, it is concluded that the most common findings of HRCT in patients with CPM include Bronchial Bonding, Ground-Glass Opacity, Emphysema and Bronchiectasis, and their most common locations were LLL, RLL, RUL, and LUL. Further studies in other centers and hospitals are needed in comparison with control groups, as well as studies on other variables such as age and gender are suggested to achieve more documentary results.
